# Effects of Mobile Mindfulness Meditation on the Mental Health of University Students: Systematic Review and Meta-analysis

**DOI:** 10.2196/39128

**Published:** 2023-01-03

**Authors:** Bin Chen, Ting Yang, Lei Xiao, Changxia Xu, Chunqin Zhu

**Affiliations:** 1 Department of Nursing Affiliated Hospital of Nanjing University of Chinese Medicine Nanjing China

**Keywords:** digital health, mobile mindfulness meditation, mental health, university students, meta-analysis

## Abstract

**Background:**

Mobile mindfulness meditation (MMM) is a mindfulness meditation intervention implemented using mobile devices such as smartphones and apps. MMM has been used to help manage the mental health of university students.

**Objective:**

This study aims to evaluate the effectiveness of MMM on the mental health of university students in the areas of stress, anxiety, depression, mindfulness, well-being, and resilience.

**Methods:**

We conducted a systematic review and meta-analysis of the effectiveness of MMM on the mental health of university students. This study followed the PRISMA (Preferred Reporting Items for Systematic Reviews and Meta-Analyses) guidelines. An electronic literature search was performed on PubMed, Web of Science, EBSCO, Cochrane Library, and Embase databases, from inception to July 16, 2021. This study was conducted to identify studies that reported the effects of MMM on the primary outcomes including stress, anxiety, and depression, and on the secondary outcomes including mindfulness, well-being, and resilience. Two reviewers retrieved articles, evaluated quality, and extracted data independently. The methodological quality of the selected studies was determined using the Cochrane criteria for risk-of-bias assessment. Standardized mean differences (SMDs) for continuous outcomes and risk ratios for dichotomous outcomes were calculated. Sensitivity analyses and subgroup analyses were performed for results with high heterogeneity. The RevMan version 5.3 was used to perform meta-analysis.

**Results:**

A total of 10 studies, including 958 university students, were selected for this meta-analysis. Results of the primary outcome showed that the MMM groups were more effective than the control groups in decreasing stress (SMD –0.41, 95% CI –0.59 to –0.23; *P*<.001) and alleviating anxiety (SMD –0.29, 95% CI –0.50 to –0.09; *P*=.004). However, there was no difference between the MMM groups and the control groups in depression (SMD –0.14, 95% CI –0.30 to 0.03; *P*=.11). The use of either waitlist control or traditional face-to-face intervention in the control group was identified as the source of heterogeneity. Specifically, the waitlist control subgroup (SMD –0.33, 95% CI –0.53 to –0.13; *P*=.002) was superior when compared with the face-to-face subgroup (SMD 0.29, 95% CI –0.01 to 0.59; *P*=.06). Results of the secondary outcome showed that the MMM groups were more effective than the control groups in enhancing well-being (SMD 0.30, 95% CI 0.11-0.50; *P*=.003) and improving mindfulness (SMD 2.66, 95% CI 0.77-4.55; *P*=.006). Whether commercial sponsorship was obtained was considered as the source of heterogeneity. The “without company support” group (SMD 17.60, 95% CI 11.32-23.87; *P*<.001) was superior to the “with company support” group (SMD 1.17, 95% CI –0.82 to 3.15; *P*=.25) in raising the level of mindfulness. However, there was no difference between the MMM and control groups in resilience (SMD –0.06, 95% CI –0.26 to 0.15; *P*=.59). The evidence level of the results from the 10 studies was determined to be moderate to low.

**Conclusions:**

MMM was an effective method to reduce stress and anxiety, and to increase the well-being and mindfulness of university students. However, further studies are needed to confirm our findings.

**Trial Registration:**

PROSPERO International Prospective Register of Systematic Reviews CRD42022303585; https://www.crd.york.ac.uk/prospero/display_record.php?RecordID=303585

## Introduction

According to the World Health Organization (WHO), mental health is “a state of well-being in which the individual realizes his or her own abilities, can cope with the normal stresses of life, can work productively and fruitfully, and is able to make a contribution to his or her community” [1]. More than 50% of university students have mental health problems [2], such as stress, anxiety, or depression [3]. Common factors associated with mental health problems are age, specifically around 18 years [4]; distance from parents and friends [5]; class learning-extracurricular activity balance [6]; and economic situation [7]. Persistent mental health problems can lead to a series of physical and psychological issues, such as sleep disorders, changes in eating habits, alcohol addiction, academic disruption [8], and suicide attempts [9]. Poor mental health among college students has become an urgent social problem that the public must focus on and solve [10].

Mindfulness meditation is a general term for various types of psychotherapy, with mindfulness at the core [11]. Mindfulness meditation is widely used to alleviate or treat emotional and psychological problems, such as anxiety, depression, compulsion, and impulsiveness [12]. Several studies have proved that mindfulness meditation is effective in reducing mental health problems such as stress and burnout [13-15]. However, as mindfulness meditation may be both time-consuming and costly, while university students may perceive the practice as a sense of stigma, most university students with mental health issues have a negative attitude toward mindfulness meditation and may be reluctant about receiving treatment [16].

In recent years, with the popularity of smartphones and development of apps [17], mindfulness meditation intervention based on smartphone apps emerged [18]. Mobile mindfulness meditation (MMM) is defined as a mindfulness meditation intervention implemented via mobile devices such as smartphones and apps, instead of face-to-face interaction. With advantages such as accessibility, absence of time and space constraints, low or no cost, and privacy [19], MMM was reported to demonstrate similar or better effects than the traditional face-to-face intervention [20,21]. Thus, MMM has attracted considerable attention for improving the mental health of university students.

Nevertheless, the effects of MMM on the mental health of university students are controversial. Some studies showed that MMM can reduce stress among university students [22,23], whereas others presented contrasting results [24,25]. Several researchers concluded that MMM may raise individuals’ level of mindfulness [23,26], whereas others suggested no such improvement [24,27]. Although MMM demonstrates potential in solving mental health problems, adequate evidence confirming the effects of MMM on the mental health of university students is lacking.

To the best of our knowledge, a meta-analysis of the effects of MMM on the mental health of university students has yet to be conducted. Few meta-analyses on the effects of MMM on the mental health of university students were conducted in previous studies. Rathbone and Prescott [28] found that MMM can significantly improve mental health; however, their study population included various population groups and was not just limited to university students with mental health problems. Regehr et al [29] reported that various types of cognitive, behavioral, and mindfulness interventions, rather than only the MMM intervention, are effective in reducing mental health problems among university students. Therefore, determining the effectiveness of MMM in reducing mental health problems among university students is essential. In this study, we aim to systematically evaluate the effects of MMM on stress, anxiety, depression, mindfulness, well-being, and resilience among university students.

## Methods

### Study Design

This study was conducted according to the PRISMA (Preferred Reporting Items for Systematic Reviews and Meta-Analyses; Multimedia Appendix 1) guidelines [30,31] and AMSTAR 2 (A Measure Tool to Assess Systematic Reviews 2) [32].

### Search Strategy

A total of 5 digital databases were searched systematically, namely, PubMed, Web of Science, EBSCO, the Cochrane Library, and Embase, from inception to July 16, 2021. Language was restricted to English. After reading a number of documents, a search strategy combining MeSH (Medical Subject Headings) terms and free words was developed. The search strategy for the 5 electronic databases is listed in Multimedia Appendix 2. Irrelevant literature was removed by reading the article titles and abstracts, and the retained literature was further screened by reading the full text. In addition, the references of each included study were retrieved manually. Two researchers (BC and TY) conducted the literature search independently, and disagreements were resolved through discussions. EndNote X9 (Clarivate) was used to import and manage the selected literature.

### Inclusion Criteria

The inclusion criteria were based on the PICOS method, as follows:

- P (population): University students with mental health problems.
- I (intervention): Various types of MMM such as the “Calm” and “Headspace” apps.
- C (comparison): Traditional mindfulness meditation, such as face-to-face therapy, waitlist control, and sham meditation.
- O (outcome): A total of 6 outcome indices were analyzed in the meta-analysis (ie, stress, anxiety, depression, well-being, mindfulness, and resilience). Stress was defined as a cognitive and behavioral experience process composed of psychological stressors and psychological stress response. Anxiety was defined as an emotional reflection of someone’s serious deterioration of the value characteristics of reality or future things. Depression was defined as a psychological disease characterized by continuous and long-term low spirits. Well-being was defined as a subjective feeling that people are satisfied with their ideal life. Mindfulness was defined as perceiving in a specific way, that is, on purpose, in the present moment, and nonjudgmentally. Resilience was defined as the ability of individuals to recover from negative experiences and flexibly adapt to the changing external environment.
- S (study design): Randomized controlled trials (RCTs) and a quasi-experimental design were used.

### Exclusion Criteria

The exclusion criteria were as follows: (1) studies not about mobile-based mindfulness meditation intervention, (2) conference articles, (3) repeated articles, and (4) studies with no original data.

### Data Extraction

Data were extracted from the selected studies and entered separately into a prefabricated form by 2 reviewers (BC and TY). Disagreements were resolved by consulting the third author (CZ). The information displayed in the form included the following: the name(s) of author(s), publication year, country, study design, sample size, experimental group intervention regimen(s), control group method(s), time interval, outcome measurement(s), and outcomes.

### Quality Assessment

The quality of each included study was assessed according to the Cochrane Handbook for Systematic Reviews of Interventions 5.1.0 [33], which includes 7 criteria, namely, random sequence generation, allocation concealment, blinding of participants and personnel, blinding of outcome assessment, incomplete data outcomes, selective outcome reporting, and other biases. Each criterion was graded as having a high risk of bias, an unclear risk of bias, or a low risk of bias. A funnel plot was used to quantify the extent of the publication bias.

The research quality was assessed by applying the GRADE (Grading of Recommendations Assessment, Development, and Evaluation) approach [34] and calculating the between-rater agreement coefficient. The *κ* coefficients were classified according to the study of Landis and Koch [35] as follows: 0.0-0.20=slight agreement, 0.21-0.40=fair agreement, 0.41-0.60=moderate agreement, 0.61-0.80=substantial agreement, and 0.81-1.00=nearly perfect agreement [36].

Two reviewers (BC and TY) independently conducted the quality assessment. Any dissenting opinion was resolved through discussion, and issues that could not be resolved were addressed by the third author (CZ).

### Statistical Analysis

The meta-analysis was conducted using RevMan 5.3 software (The Cochrane Collaboration). The weighted mean difference model was used to analyze the continuous data if all the outcomes were measured using identical methods; otherwise standardized mean difference (SMD) was used. An *I*^2^ test was conducted to assess the degree of heterogeneity of included studies, and *I*^2^>50% indicated significant heterogeneity according to the Cochrane Handbook. The *P* and *I*^2^ values were used to determine the model to choose. A fixed-effect model was chosen if *P*>.1 and *I*^2^<50%, whereas a random-effect model was selected if *P*<.1 and *I*^2^>50%. In addition, a sensitivity analysis through the leave-one-out method and a subgroup analysis were performed on studies with significant heterogeneity. All effective quantities were expressed by a 95% CI, and *P*<.05 defined the statistical significance.

## Results

### Search Findings

**Figure 1:**
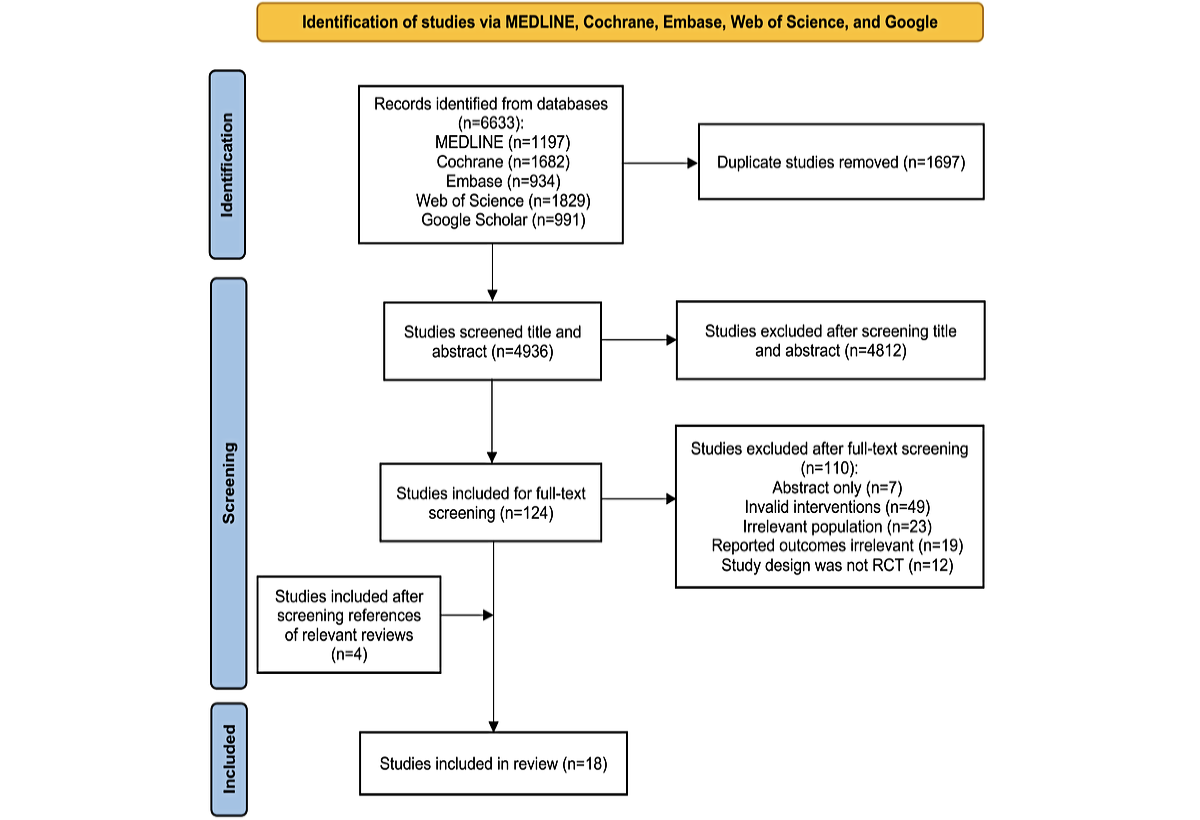
Studyflow diagram.

A total of 2695 records were identified for retrieval, specifically PubMed (n=2223), Web of Science (n=178), EBSCO (n=4), the Cochrane Library (n=222), Embase (n=66), and additional records from the references provided in the included studies (n=2). After the duplicates were removed, 2030 articles remained. A total of 1982 records were excluded after a thorough review of the titles and abstracts. Of the remaining 48 records, 38 were removed after a full-text screening. Finally, 10 articles [22-27,37-40] were selected for the systematic review and meta-analysis. The study flow diagram of the selection process is shown in Figure 1.

### Study Characteristics

The 10 included studies were published between 2018 and 2021 (all within the past 5 years). A total of 3 studies were conducted in the United States [23,24,37], with 1 each in Iran [25], Canada [38], Ireland [39], Spain [26], United Kingdom [40], Finland [27], and Germany [22]. The RCT design was employed for 9 articles [22-24,26,27,37-40], whereas the quasi-experimental study design was used for 1 article [25]. A total of 958 university students were included in this review who were allocated to an experimental group (n=461) and a control group (n=497). MMM involves mindfulness intervention delivered via apps installed on smartphones, and mainly focuses on college students. This intervention method was the only strategy used for the mindfulness meditation intervention in the experimental group. Although the MMM intervention for the control group was conducted using various types of smartphones or apps, all of these were the MMM method. Traditional face-to-face mindfulness meditation or waitlist control was used for the students in the control group. The time interval of the study ranged from 30 days to 8 weeks. All outcomes were completely patient reported. The main characteristics of the selected studies are shown in Table 1.

**Table 1:** Characteristics of included studies.

Study	Country	Design	Sample size, n	T^a^/C^b^, n	Intervention	Control	Time interval	Outcome measures and their values (T/C), mean (SD)
Borjalilu et al [25]	Iran	Quasi-experimental study	48	28/20	Applied the “Aramgar” stress management app	Face-to-face therapy only	6 weeks	Depression: 12.22 (5.34)/9.45 (5.67) Stress: 11.23 (4.34)/10.61 (4.73) Anxiety: 11.43 (4.57)/10.34 (4.34)
Champion et al [37]	United States	RCT^c^	74	38/36	The self-guided mindfulness meditation app “Headspace”	Waitlist control	30 days	Resilience: 76.97 (10.53)/76.39 (11.92)
Huberty et al [23]	United States	RCT	88	41/47	The “Calm” smartphone app	Waitlist control	8 weeks	Stress: 16.15 (6.16)/20.02 (6.16) Mindfulness: 129.20 (18.32)/111.07 (18.31)
Lee and Jung [38]	Canada	RCT	163	77/86	The “DeStressify” app	Waitlist control	8 weeks	Stress: 17.8 (6.2)/19.8 (6.7) Anxiety: 40.1 (12.1)/43.4 (13.2) Depression: 6.4 (3.9)/7.4 (4.7)
Noone and Hogan [39]	Ireland	RCT	91	43/48	The “Headspace” app	Sham meditation group	6 weeks	Well-being: 50.28 (6.91)/49.98 (6.58)
Orosa-Duarte et al [26]	Spain	RCT	61	31/30	The “REM Volver a casa” (Mindfulness-Based Emotion Regulation. Going Home”) app	Did not receive any intervention	8 weeks	Anxiety: 20.48 (12.51)/26.77 (12.57) Mindfulness: 140.35 (20.44)/123.70 (21.25)
Ponzo et al [40]	United Kingdom	RCT	116	55/61	The “BioBase” app	Waitlist control	4 weeks	Anxiety: 46.31 (11.32)/51.33 (10.35) Well-being: 42.15 (9.02)/40.51 (8.64) Depression: 8.71 (4.45)/9.85 (5.38)
Raevuori et al [27]	Finland	RCT	124	63/61	The Meru Health Program via smartphone	Treatment as usual	8 weeks	Depression: 9.89 (6.30)/8.57 (6.04) Mindfulness: 73.19 (11.87)/72.59 (11.40) Resilience: 81.82 (11.24)/82.32 (10.78)
Harrer et al [22]	Germany	RCT	105	40/65	An app-based intervention with feedback on demand (StudiCare Stress)	Waitlist control	7 weeks	Stress: 7.43 (2.93)/9.49 (3.06) Depression: 15.88 (8.85)/21.47 (8.96) Well-being: 11.93 (5.03)/9.36 (4.35) Resilience: 5.38 (1.85)/5.05 (1.97)
Yang et al [24]	United States	RCT	88	45/43	The “Headspace” app	Waitlist control	30 days	Stress: 17.08 (6.02)/19.30 (s5.63) Mindfulness: 25.69 (4.96)/24.35 (5.27) Well-being: 77.10 (11.97)/71.38 (12.37)

^a^T: test group.

^b^C: control group.

^c^RCT: randomized controlled trial.

### Quality of Studies

**Figure 2:**
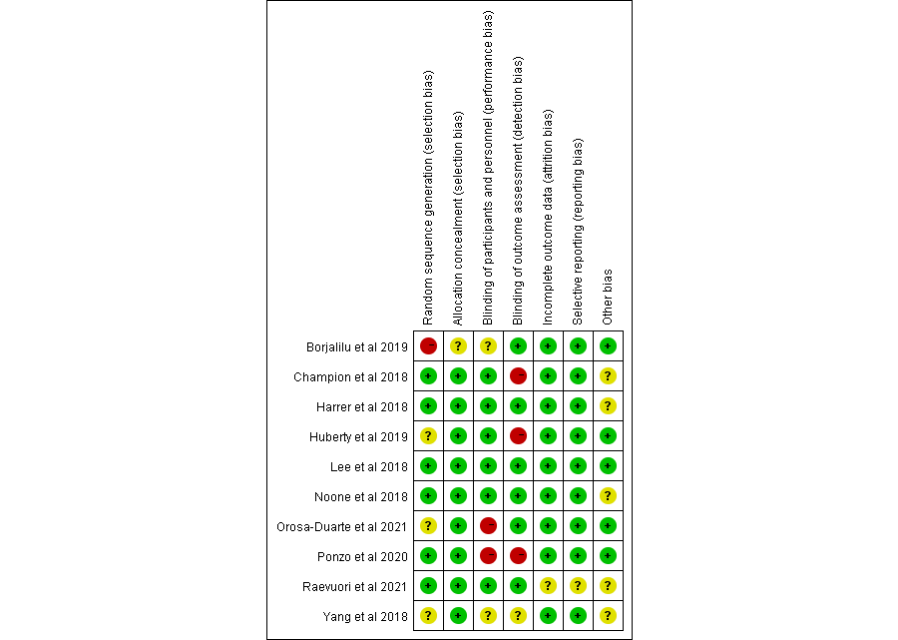
Risk of bias of each included study. See also [22,23-27,36-40].

**Figure 3:**
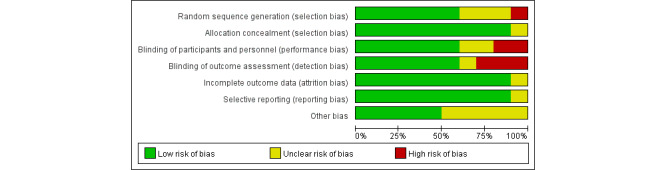
Overall risk of bias analysis of included studies.

Based on the Cochrane Handbook for Systematic Reviews of Interventions criteria, the bias assessment of the selected articles is shown in Figures 2 and 3. Six studies [22,27,37-40] reported randomized methods in detail, 3 studies [23,24,26] used randomization without stating a specific random scheme, and the remaining study [25] did not use random grouping. All studies except 1 [25] described allocation concealment. Moreover, 6 studies [22,23,27,37-39] used blinding methods for the participants and personnel, and 6 studies [22,25-27,38,39] used outcome assessment blinding. All the studies except 1 [27] identified incomplete outcome data and selective reporting as low risks. Furthermore, 5 studies [23,25,26,38,40] reported other bias as low risks. This review did not draw funnel plots owing to the insufficient number of studies for each outcome.

### Meta-analysis Findings

#### Stress

**Figure 4:**
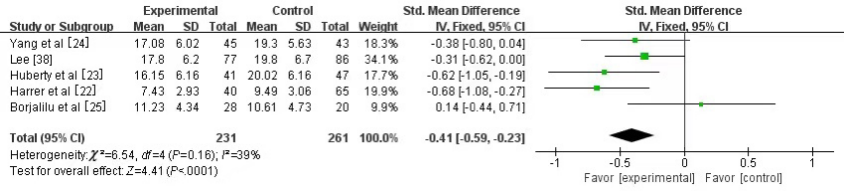
Forest plot of mobile mindfulness meditation on stress. See also [22-25,37].

A total of 5 studies [22-25,38] involving 690 university students assessed stress as the outcome. Among the 5 studies, 4 [22-24,38] used the Perceived Stress Scale (PSS) [41], and 1 [25] used the 21-item Depression, Anxiety, and Stress Scale (DASS-21) [42]. Owing to the different measurement tools used in the studies, SMD was chosen for the meta-analysis. A fixed-effect model was selected for the statistical analysis, as low heterogeneity was observed between the studies (*I*^2^=39%, *P*=.16). It was found that stress decreased significantly in the experimental group (SMD –0.41, 95% CI –0.59 to –0.23; *P*<.001) compared with that in the control group (Figure 4).

#### Anxiety

**Figure 5:**
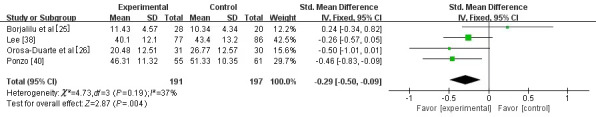
Forest plot of mobile mindfulness meditation on anxiety. See also [25,26,37,39].

A total of 4 studies [25,26,38,40] involving 617 university students assessed anxiety as the outcome. Among the 4 studies, 3 [26,38,40] used the State-Trait Anxiety Inventory (STAI) [43], and 1 [25] used the DASS-21 [42]. SMD was chosen owing to the different types of measurement tools used in the studies. A fixed-effect model was selected for the statistical analysis, as low heterogeneity was observed between the studies (*I*^2^=37%, *P*=.19). It was found that anxiety decreased significantly in the experimental group (SMD –0.29, 95% CI –0.50 to –0.09; *P*=.004) compared with that in the control group (Figure 5).

#### Depression

**Figure 6:**
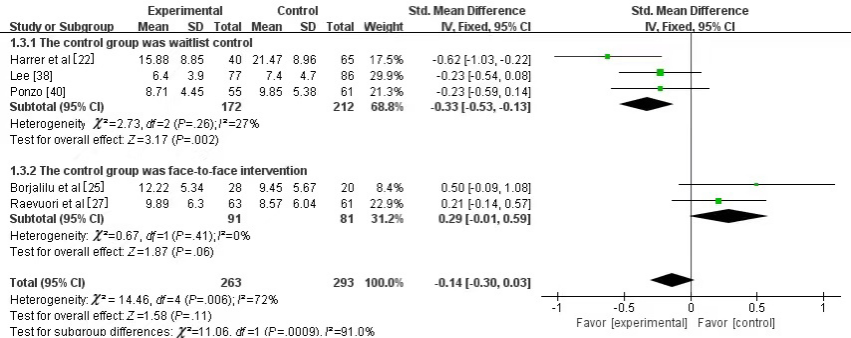
Forest plot of mobile mindfulness meditation on depression. See also [22,25,27,37,39].

A total of 5 studies [22,25,27,38,40] involving 556 university students assessed depression. Among the 5 studies, 2 [27,40] used the 9-item Patient Health Questionnaire (PHQ-9) [44] and the other 3 [22,25,38] used the Center for Epidemiological Studies’ Depression Scale (CES-D) [45], the Quick Inventory of Depressive Symptomatology Self-Report (QIDS-SR) [46], and the DASS-21 [42] separately. Owing to the different measurement tools used in the studies, SMD was chosen for the meta-analysis. No statistical significance was found between the experimental and control groups (SMD –0.14, 95% CI –0.30 to 0.03; *P*=.11). Moreover, significant heterogeneity (*I*^2^=72%, *P*=.006) was observed between the 5 studies. Sensitivity analysis was performed on the outcome, but no individual study was found to qualitatively affect the results. Subgroup analysis was conducted to determine the differences between the studies. The use of either waitlist control or traditional face-to-face intervention in the control group was identified as the source of heterogeneity: specifically, the waitlist control subgroup (SMD –0.33, 95% CI –0.53 to –0.13; *P*=.002) versus the face-to-face subgroup (SMD 0.29, 95% CI –0.01 to 0.59; *P*=.06). The results showed that the effects of MMM and traditional face-to-face mindfulness meditation on depression were similar (Figure 6).

#### Well-being

**Figure 7:**
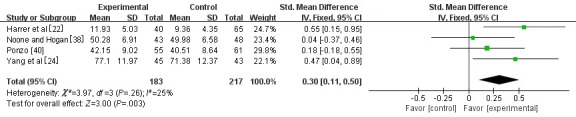
Forest plot of mobile mindfulness meditation on well-being. See also [22,24,38,39].

Four studies [22,24,39,40] involving 400 university students assessed anxiety as the outcome. Among the 4 studies, 2 [39,40] used the Warwick-Edinburgh Mental Well-Being Scale (WEMWBS) [47], and the remaining 2 [22,24] used the WHO-Five Well-Being Index (WHO-5) [48] and the General Well-being Schedule (GWBS) [49]. SMD was chosen owing to different types of measurement tools used in the studies. A fixed-effect model was selected for the statistical analysis, as small heterogeneity among studies was observed between the studies (*I*^2^=25%, *P*=.26). It was found that well-being increased significantly in the experimental group (SMD 0.30, 95% CI 0.11-0.50; *P*=.003) compared with the control group (Figure 7).

#### Mindfulness

**Figure 8:**
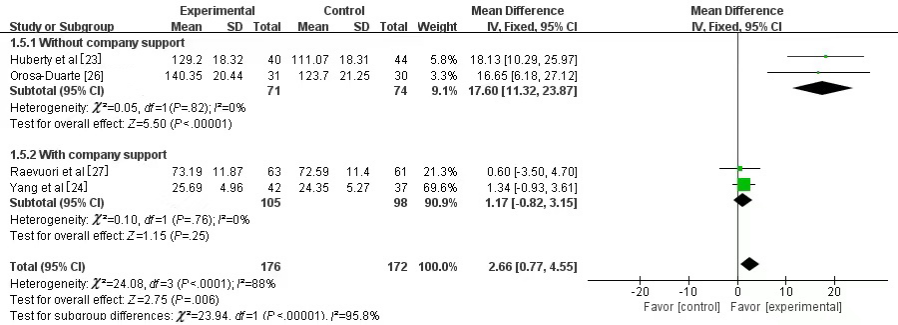
Forest plot of mobile mindfulness meditation on mindfulness. See also [23-25,27].

Four studies [23,24,26,27] involving 348 university students assessed mindfulness. All 4 studies used the Five Factor Mindfulness Questionnaire (FFMQ) [50]. Mean difference was chosen for the meta-analysis, and it was found that mindfulness increased significantly in the experimental group (SMD 2.66, 95% CI 0.77-4.55; *P*=.006) compared with the control group. However, significant heterogeneity (*I*^2^=88%, *P*<.001) was observed between the 4 studies. Sensitivity analysis was performed on the outcome, but no individual study was found to qualitatively affect the results. Subgroup analysis was conducted to determine the differences between the studies. Support from a company may be the source of the heterogeneity, specifically the “without company support” subgroup (SMD 17.60, 95% CI 11.32-23.87; *P*<.001) versus the “with company support” subgroup (SMD 1.17, 95% CI –0.82 to 3.15; *P*=.25). The results showed that MMM without company support significantly improved mindfulness (*P*<.001; Figure 8).

#### Resilience

**Figure 9:**
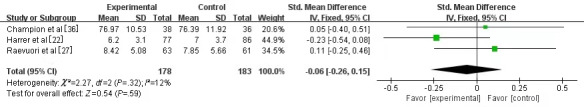
Forest plot of mobile mindfulness meditation on resilience. See also [22,27,36].

A total of 3 studies [22,27,37] involving 302 university students assessed resilience as the outcome. Two studies [27,37] used the Wagnild Resilience Scale (WRS) [51] and 1 [22] used the Connor-Davidson Resilience Scale (CD-RISC) [52]. SMD was selected owing to different types of measurement tools used in the studies. A fixed-effect model was selected for the statistical analysis, as low heterogeneity was observed between the studies (*I*^2^=12%, *P*=.32). No statistical significance was found between the experimental group and the control group (SMD –0.06, 95% CI –0.26 to 0.15; *P*=.59; Figure 9).

#### Quality of Evidence

The results showed that the quality of evidence ranged from moderate to low. The between-rater *κ* coefficient was 100% (*P*=.001), indicating perfect between-rater agreement during the evaluation process. The quality of the evidence is summarized in Table 2.

**Table 2:** GRADE^a^ summary of findings for all outcomes.

Outcomes	Effect estimate (95% CI)	Studies, n	Participants, n	*I*^2^ (%)	Quality of evidence (reasons for downgrading)
Stress	–0.41 (–0.59 to –0.23)	5	690	39	⊕⊕^b^Low (inconsistency; publication bias)
Anxiety	–0.29 (–0.50 to –0.09)	4	617	37	⊕⊕⊕^c^Moderate (inconsistency)
Depression	–0.14 (–0.30 to 0.03)	5	556	72	⊕⊕Low (inconsistency; publication bias)
Well-being	0.30 (0.11 to 0.50)	4	400	25	⊕⊕Low (inconsistency; publication bias)
Mindfulness	2.66 (0.77 to 4.55)	4	348	88	⊕⊕⊕Moderate (publication bias)
Resilience	–0.06 (–0.26 to 0.15)	3	302	12	⊕⊕Low (inconsistency; publication bias)

^a^GRADE: Grading of Recommendations Assessment, Development, and Evaluation.

^b^⊕⊕ means there are 2 reasons for downgrading.

^c^⊕⊕⊕ means there are 3 reasons for downgrading.

## Discussion

### Principal Findings

A meta-analysis was conducted on 10 studies to assess the effects of MMM on stress, anxiety, depression, mindfulness, well-being, and resilience among 958 university students. All the studies used MMM in the experimental group and traditional face-to-face mindfulness meditation or waitlist control in the control group. Funnel plots are typically used to assess publication bias when at least ten studies are used for the meta-analysis [53]; however, a funnel plot was not generated in this study, as the number of studies for each outcome was less than 10. The time interval of the study ranged from 30 days to 8 weeks; however, we did not find significant difference between each included studies, which is likely due to the limited study number. We believe that with the increase of the number of studies, this phenomenon will receive more general attention. Overall, a varying quality of evidence was observed for the effects of MMM on the mental health of university students. Specifically, for stress, a low quality of evidence was observed for the finding that MMM significantly reduced stress among university students. For anxiety, a moderate quality of evidence was observed for the result that MMM significantly decreased anxiety in the experimental group compared with the control group. In terms of depression, the low quality of evidence result indicated no statistical significance between the experimental and control groups. With regard to well-being, a low quality of evidence was observed for the outcome that university students in the MMM group had higher well-being than those in the control group. For mindfulness, a moderate quality of evidence was found on the finding that MMM positively enhanced the university students’ mindfulness. Finally, regarding resilience, a low quality of evidence indicated no statistical significance between the experimental and control groups.

### Primary Outcome

#### Overview

The primary outcome of our study was the effectiveness of MMM in reducing stress, anxiety, and depression among university students. These 3 psychological problems were reported as significant factors influencing students’ academic performance and physical health, such as back pains, headaches, and irritable bowel syndrome [54]. Thus, developing effective mental health management strategies to address the aforementioned psychological issues is essential [23].

#### Stress

Stress is an individuals’ natural reflection in the face of tension, which may lead to worry, restlessness, among others [55]. This study showed a significant reduction in stress among university students in the MMM intervention group (SMD –0.41, 95% CI –0.59 to –0.23; *P*<.001), which is similar to the findings of previous studies [22,23,56]. Possible reasons for this outcome exist. For example, previous studies proved the positive effectiveness of traditional mindfulness therapy in reducing stress among university students [57,58]. Mindfulness meditation through smartphone apps may improve muscle relaxation and diaphragm breathing, focus attention on different parts of the body, raise noncritical awareness of events occurring continuously, and enhance self-reflection [59]. In addition, MMM requires less resources (in terms of cost, personnel, or buildings), as it can be easily accessed through smartphones, regardless of economic and other constraints [60]. In particular, MMM involves minimal time and space constraints, as university students can just turn on their smartphones to receive mindfulness treatment anytime and anywhere at their convenience, instead of setting a fixed time and place for unified concentration, such as in traditional methods. Such advantages can help students obtain increased and convenient treatment, which may be a highly practical way to reduce stress [23].

#### Anxiety

Anxiety refers to an unpleasant complex emotional state, such as tension, uneasiness, and worry, caused by imminent or possible danger or threat [61]. This study revealed that university students in the experimental group were able to lower their anxiety level compared with those in the control group (SMD –0.29, 95% CI –0.50 to –0.09; *P*=.004), which is consistent with previous findings [40,58]. The possible causes were analyzed as follows: university students often feel stigmatized when they reveal having mental issues, and may be reluctant to seek treatment in the school hospital or psychological counseling center from fear of being seen by classmates or teachers, who may label them as having mental health problems, which can further aggravate their anxiety and other problems [62]. Through mobile devices, students can receive treatment remotely, thus avoiding the embarrassment of face-to-face contact [38]. Intervention through mobile devices may have several advantages, such as usefulness, convenience, and ease of use; additionally, it is more popular among and easily accepted by university students [63]. This improved engagement and accessibility in anxious individuals who may prefer apps to face-to-face group mindfulness programs. Interestingly, a previous study reported that smartphone-aided psychological treatments can decrease anxiety among users [64], which may be another reason for the outcomes.

#### Depression

The main clinical characteristics of depression are manifested mainly as depressed moods, slow thinking, reduced language and movement, and retardation [65]. This study showed no statistical difference in depression between university students in the experimental group and those in the control group. Heterogeneity was detected between the studies (*I*^2^=72%, *P*=.006), whereas the sensitivity analysis indicated that no single study qualitatively affected the combined results. Subgroup analysis was performed to identify the potential causes of the heterogeneity. For the experimental design process, in the case of similar interventions in the experimental group, 3 of the 5 studies set the control group as a waitlist control, and the other 2 studies used traditional face-to-face mindfulness meditation. Thus, it can be concluded that the “face-to-face mindfulness meditation” group (SMD 0.29, 95% CI –0.01 to 0.59; *P*=.06) was superior to the “waitlist control” group (SMD –0.33, 95% CI –0.53 to –0.13; *P*=.002) in terms of reduced symptoms of depression. This result is in line with previous findings [25,27]. A reason for this outcome maybe that MMM and traditional mindfulness meditation methods can effectively alleviate the depressive symptoms of university students and have the same effect size [25,27]; thus, no significant difference was observed between the MMM and traditional intervention control groups. Meanwhile, no intervention measures were used in the waitlist control group; thus, the depressive symptoms of university students in this group did not change significantly [22]. Therefore, a significant difference existed between the experimental and control groups.

### Secondary Outcome

#### Overview

The secondary outcome of our study was the effectiveness of MMM in increasing well-being, mindfulness, and resilience among university students. The 3 outcomes had a close positive correlation with mental health [66]. Improving well-being, mindfulness, and resilience can help university students to effectively deal with mental distress, such as stress, anxiety, and depression [67].

#### Well-being

Well-being refers to a series of joyful emotions subjectively generated by individuals based on their sense of satisfaction and security [68]. Compared with university students in the control group, MMM may strengthen the well-being of those in the experimental group (SMD 0.30, 95% CI 0.11-0.50; *P*=.003), which is in line with the findings of previous studies [22,24]. A possible reason is that the advantage of the MMM intervention is the rigorous smartphone intervention, because the standardized guidance scheme and objective compliance measures of the participants in the experimental group were provided by apps; thus, the intervention method was homogeneous [1]. Likewise, a recent meta-analysis showed that 67% of positive psychological interventions are conducted in the form of self-help. This form is similar to the advantage offered by MMM based on smartphone apps, as university students could choose an intervention performed based on their needs [69]. Smartphone-based self-intervention programs allow university students to avoid certain risks and restrictions related to traditional methods, such as time, resource, flexibility, accessibility, and availability constraints [70]. Such programs also meet the requirements of the WHO, especially the use of electronic and mobile health technologies [71].

#### Mindfulness

Mindfulness is perceived in a specific way, that is, conscious awareness, living in the present, and not making judgments [72]. Our study revealed a significant improvement in the mindfulness of university students in the MMM intervention group. Heterogeneity was detected between the studies (*I*^2^=88%, *P*<.001), and the sensitivity analysis suggested that no single study qualitatively affected the combined results. Subgroup analysis was performed to identify the potential causes of the heterogeneity. In the design process, 2 of the 4 studies were funded or technically guided by professional companies, whereas the other 2 did not obtain business funding or guidance. Thus, it can be concluded that the “without company support” group (SMD 17.60, 95% CI 11.32-23.87; *P*<.001) was superior to the “with company support” group (SMD 1.17, 95% CI –0.82 to 3.15; *P*=.25) in raising the level of mindfulness. This result is in line with previous findings [23,26]. Besides, the results of the studies funded or technically guided by companies may have risks of bias [33]. Thus, caution should be exercised when interpreting such findings. Research supports this study’s idea that a significant advantage of MMM is the objective recording of the number of meditation sessions completed by university students by an app instead of self-reporting [73].

#### Resilience

Changes in psychological resilience among university students were also observed. Resilience refers to an individuals’ ability to recover from negative experiences and adapt flexibly to the changing external environment [74]. The results indicated no statistical difference between the 2 groups (SMD –0.06, 95% CI –0.26 to 0.15; *P*=.59), which are in line with those of previous studies [22,27,37]. A possible reason for this outcome may be that resilience-based interventions emphasize individuals’ and community members’ strength and ability to recover from physical, emotional, or environment stress [75]. A positive correlation was observed between psychological resilience and education level [76]. University students have a high education level; therefore, it was assumed in this study that university students had high psychological resilience before seeking the MMM intervention.

### Strengths and Limitations

This meta-analysis study has several strengths. First, this is the first study to assess the effects of MMM on the mental health of university students. It also represents the future development direction of the mindfulness intervention to university students. Second, all studies were published in the last 5 years, which indicates that this is a whole new research field. Third, to assess the effects of the MMM intervention, we assessed 6 outcomes, including stress, anxiety, depression, well-being, mindfulness, and resilience, which might provide reference for mental health management of university students.

Limitations to this study were as follows: first, selected articles were limited to the English language, which may lead to the lack of some high-quality articles in non-English languages. Second, different smartphones and apps were used in each study, which may contribute to heterogeneity. Third, regarding the 6 outcomes, different measurements were used for each outcome, which may have had an impact on the bias of the results. Lastly, due to the limited number of included studies, we did not classify different time points of outcome assessment used in studies and consider how this may influence the final results.

### Relevance for Clinical Practice

This review study presents evidence regarding the effectiveness of MMM in improving the mental health problems of university students. With the serious mental health status of university students [2], this provides a new idea and a method to improve such symptoms. At the same time, it also provides some reference for the MMM research field to improve mental health.

### Conclusions

This meta-analysis investigated the effectiveness of MMM on stress, anxiety, depression, well-being, mindfulness, and resilience of university students. In general, the effectiveness of MMM on the mental health of university students was superior to that of traditional mindfulness meditation. There were positive effects of MMM in decreasing stress, alleviating anxiety, enhancing well-being, and improving mindfulness of university students. Concerning the outcomes depression and resilience, the MMM method contributed equally to the traditional face-to-face mindfulness meditation method. Therefore, in view of the results of meta-analysis, we cautiously proposed that MMM was an effective method to reduce stress and anxiety, and to increase the well-being and mindfulness of university students. However, due to the limited number of studies that have examined the effectiveness of MMM on mental health, further studies are needed to confirm our findings.

## Appendix

Multimedia Appendix 1 PRISMA (Preferred Reporting Items for Systematic Reviews and Meta-Analyses) 2020 checklist.

Multimedia Appendix 2 Search strategies.

## Abbreviations

AMSTAR 2: A Measure Tool to Assess Systematic Reviews 2
CD-RISC: Connor-Davidson Resilience Scale
CES-D: Center for Epidemiological Studies’ Depression Scale
DASS-21: 21-item Depression, Anxiety, and Stress Scale
FFMQ: Five Factor Mindfulness Questionnaire
GRADE: Grading of Recommendations Assessment, Development, and Evaluation
GWBS: General Well-being Schedule
MeSH: Medical Subject Headings
MMM: mobile mindfulness meditation
PHQ-9: 9-item Patient Health Questionnaire
PICOS: population, intervention, comparison, outcome, study design
PRISMA: Preferred Reporting Items for Systematic Reviews and Meta-Analyses
PSS: Perceived Stress Scale
QIDS-SR: Quick Inventory of Depressive Symptomatology Self-Report
RCT: randomized controlled trial
STAI: State-Trait Anxiety Inventory
WEMWBS: Warwick-Edinburgh Mental Well-Being Scale
WHO: World Health Organization
WHO-5: WHO-Five Well-Being Index
WRS: Wagnild Resilience Scale
