# Supplementary material for: Effects of Mobile Mindfulness Meditation on the Mental Health of University Students: Systematic Review and Meta-analysis
Source: J Med Internet Res. 2023 Jan 3;25:e39128. doi: 10.2196/39128 (PMC9856434; doi:10.2196/39128)
Supplement: Multimedia Appendix 2 [file jmir_v25i1e39128_app2.doc]

## Multimedia Appendix 2:

## Search strategies

PubMed

#1. Mobile Applications[MeSH Terms]

#2. Cell Phone[MeSH Terms]

#3. Cell Phone Use[MeSH Terms]

#4. (((((((((((((((((((((((((mobile[Title/Abstract])) OR (mobile applications[Title/Abstract])) OR (mobile based[Title/Abstract])) OR (mobile-based[Title/Abstract])) OR (distance counseling[Title/Abstract])) OR (app[Title/Abstract])) OR (app based[Title/Abstract])) OR (app-based[Title/Abstract])) OR (software[Title/Abstract])) OR (electronic[Title/Abstract])) OR (digital[Title/Abstract])) OR (smartphon[Title/Abstract])) OR (phone[Title/Abstract])) OR (online[Title/Abstract])) OR (internet[Title/Abstract])) OR (web[Title/Abstract])) OR (ehealth[Title/Abstract])) OR (telehealth[Title/Abstract])) OR (telebased[Title/Abstract])) OR (tele-based[Title/Abstract])) OR (telemedicine[Title/Abstract])

#5. #1 OR #2 OR #3 OR #4

#6. mindfulness[MeSH Terms]

#7. (((((((mindfulness[Title/Abstract])) OR (meditation[Title/Abstract])) OR (mindfulness-based intervention[Title/Abstract])) OR (MBSR[Title/Abstract])) OR (mindfulness-based stress reduction[Title/Abstract])) OR (mindfulness-based cognitive therapy[Title/Abstract])) OR (vipassana[Title/Abstract])

#8. #6 OR #7

#9. student[MeSH Terms]

#10. (((((college[Title/Abstract])) OR (university[Title/Abstract])) OR (student[Title/Abstract])) OR (undergraduate[Title/Abstract])) OR (baccalaureate[Title/Abstract])

#11. #9 OR #10

#12. #5 AND #8 AND #11

Web of Science

#1. (‎(‎(‎(‎(‎(‎(‎(‎(‎(‎(‎(‎(‎(‎(‎(‎(‎(‎(‎(‎Subject: (‎mobile) OR Subject: (‎mobile applications)) OR Subject: (‎mobile based)) OR Subject: (‎mobile-based)) OR Subject: (‎distance counseling)) OR Subject: (‎app)) OR Subject: (‎app based)) OR Subject: (‎app-based)) OR Subject: (‎software)) OR Subject: (‎electronic)) OR Subject: (‎digital)) OR Subject: (‎smartphone)) OR Subject: (‎phone)) OR Subject: (‎online)) OR Subject: (‎internet)) OR Subject: (‎web)) OR Subject: (‎eheat)) OR Subject: (‎telehealth)) OR Subject: (‎tilebased)) OR Subject: (‎tele-based)) OR Subject: (‎telemedicine))

#2. Subject: (mindfulness) OR Subject: (meditation) OR Subject: (mindfulness-based intervention) OR Subject: (MBSR) OR Subject: (mindfulness-based stress reduction) OR Subject: (mindfulness-based cognitive therapy) OR Subject: (vipassana)

#3. Subject: (college) OR Subject: (university) OR Subject: (student) OR Subject: (undergraduate) OR Subject: (baccalaureate)

#4. Subject: (randomized controlled trial) OR Subject: (randomized) OR Subject: (randomly) OR Subject: (RCT)

#5. #1 AND #2 AND #3 AND #4

EBSCO

#1. SU mobile OR SU mobile applications OR SU mobile based OR SU mobile-based OR SU distance counseling OR SU app OR SU app based OR SU app-based OR SU software OR SU electronic OR SU digital OR SU smartphone OR SU phone OR SU online OR SU internet OR SU web OR SU ehealth OR SU telehealth OR SU telebased OR SU tele-based OR SU telemedicine

#2. SU mindfulness OR SU meditation OR SU mindfulness-based intervention OR SU MBSR OR SU mindfulness-based stress reduction OR SU mindfulness-based cognitive therapy OR SU vipassana

#3. SU college OR SU university OR SU student OR SU undergraduate OR SU baccalaureate

#4. SU randomized controlled trial OR SU randomized OR SU randomly OR SU RCT

#5. #1 AND #2 AND #3 AND #4

Cochrane:

#1. (mobile):ti,ab,kw OR (mobile applications):ti,ab,kw OR (mobile based):ti,ab,kw OR (mobile-based):ti,ab,kw OR (distance counseling):ti,ab,kw OR (app):ti,ab,kw OR (app based):ti,ab,kw OR (app-based):ti,ab,kw OR (software):ti,ab,kw OR (electronic):ti,ab,kw OR (digital):ti,ab,kw OR (smartphone):ti,ab,kw OR (phone):ti,ab,kw OR (online):ti,ab,kw OR (internet):ti,ab,kw OR (web):ti,ab,kw OR (ehealth):ti,ab,kw OR (telehealth):ti,ab,kw OR (telebased):ti,ab,kw OR (tele-based):ti,ab,kw OR (telemedicine):ti,ab,kw

#2. (mindfulness):ti,ab,kw OR (meditation):ti,ab,kw OR (mindfulness-based intervention):ti,ab,kw OR (MBSR):ti,ab,kw OR (mindfulness-based stress reduction):ti,ab,kw OR (mindfulness-based cognitive therapy):ti,ab,kw OR (vipassana):ti,ab,kw

#3. (college):ti,ab,kw OR (university):ti,ab,kw OR (student):ti,ab,kw OR (undergraduate):ti,ab,kw OR (baccalaureate):ti,ab,kw

#4. (randomized controlled trial):ti,ab,kw OR (randomized):ti,ab,kw OR (randomly):ti,ab,kw OR (RCT):ti,ab,kw

#5. #1 AND #2 AND #3 AND #4

EMBASE

#1. (mobile):ti,ab,kw OR (mobile applications):ti,ab,kw OR (mobile based):ti,ab,kw OR (mobile-based):ti,ab,kw OR (distance counseling):ti,ab,kw OR (app):ti,ab,kw OR (app based):ti,ab,kw OR (app-based):ti,ab,kw OR (software):ti,ab,kw OR (electronic):ti,ab,kw OR (digital):ti,ab,kw OR (smartphone):ti,ab,kw OR (phone):ti,ab,kw OR (online):ti,ab,kw OR (internet):ti,ab,kw OR (web):ti,ab,kw OR (ehealth):ti,ab,kw OR (telehealth):ti,ab,kw OR (telebased):ti,ab,kw OR (tele-based):ti,ab,kw OR (telemedicine):ti,ab,kw

#2. (mindfulness):ti,ab,kw OR (meditation):ti,ab,kw OR (mindfulness-based intervention):ti,ab,kw OR (MBSR):ti,ab,kw OR (mindfulness-based stress reduction):ti,ab,kw OR (mindfulness-based cognitive therapy):ti,ab,kw OR (vipassana):ti,ab,kw

#3. (college):ti,ab,kw OR (university):ti,ab,kw OR (student):ti,ab,kw OR (undergraduate):ti,ab,kw OR (baccalaureate):ti,ab,kw

#4. (randomized controlled trial):ti,ab,kw OR (randomized):ti,ab,kw OR (randomly):ti,ab,kw OR (RCT):ti,ab,kw

#5. #1 AND #2 AND #3 AND #4
